# Development and Usability of the “FORTEe Get Strong” App to Promote Physical Activity and Health Awareness in Children and Adolescents With Cancer During Intensive Treatment Using an App-Based Approach: Mixed Methods Study

**DOI:** 10.2196/75653

**Published:** 2026-04-01

**Authors:** Mareike Kuehn, Lena Wypyrsczyk, Francesca Lanfranconi, Adriana Cristina Balduzzi, Hayley Marriott, Alba Solera-Sanchez, Joachim Wiskemann, Nikolai Bauer, Nina Karguth, Barbara Heißerer, Norbert W Paul, Rodolf Mongondry, Wilhelm Bloch, Katie Rizvi, Martin Kaj Fridh, Eva S C Ramos, Carmen Fiuza-Luces, Miriam Götte, Gabriele Gauß, Filippo Spreafico, Barbara Konda, Milica Stefanović, Elias Dreismickenbecker, Marie A Neu, Joerg Faber, FORTEe Consortium, Christian Ruckes

**Affiliations:** 1 Childhood Cancer Center Mainz, University Medical Center of the Johannes Gutenberg University Mainz, Langenbeckstraße 1, Mainz, 55131, Germany, 49 6131178331; 2 Department of Sports Medicine, Prevention and Rehabilitation, Institute of Sport Science, Johannes Gutenberg-University Mainz, Mainz, Germany; 3 Fondazione Monza e Brianza per il bambino e la sua mamma, Centro Maria Letizia Verga, Monza, Italy; 4 School of Medicine and Surgery, University of Milano-Bicocca, Milan, Italy; 5 Pediatrics, Fondazione IRCCS San Gerardo dei Tintori, Monza, Italy; 6 School of Sport, Nutrition and Allied Health Professions, Oxford Brookes University, Oxford, United Kingdom; 7 Department of Medical Oncology, Working Group Exercise Oncology, a partnership between DKFZ and University Medical Center Heidelberg, Heidelberg University Hospital and National Center for Tumor Diseases, Heidelberg, Germany; 8 Concentris research management GmbH, Fürstenfeldbruck, Germany; 9 Institute for the History, Philosophy and Ethics of Medicine, University Medical Center of the Johannes Gutenberg University Mainz, Mainz, Germany; 10 Centre de Lutte Contre le Cancer, Centre Léon Bérard, Lyon, France; 11 Department of Molecular and Cellular Sport Medicine, German Sport University Cologne, Cologne, Germany; 12 Youth Cancer Europe, Cluj-Napoca, Romania; 13 Department of Pediatrics and Adolescent Medicine, Rigshospitalet, Copenhagen University Hospital, Copenhagen, Denmark; 14 Department of Sport Sciences, Faculty of Medicine, Health and Sports, Universidad Europea de Madrid, Madrid, Spain; 15 Research Institute Hospital 12 de Octubre, Madrid, Spain; 16 West German Cancer Center, Essen University Hospital, Essen, Germany; 17 Hematology and Oncology, Clinic for Pediatrics III, Essen University Hospital, Essen, Germany; 18 Oncology Unit, IRCCS Istituto Giannina Gaslini, Genoa, Italy; 19 Pediatric Oncology Unit, Fondazione IRCCS Istituto Nazionale dei Tumori, Milan, Italy; 20 Forma 3D Ltd, Ljubljana, Slovenia; 21 Ljubljana University Medical Centre, Ljubljana, Slovenia; 22 See Acknowledgments

**Keywords:** childhood cancer, exercise, oncology, serious game, gamification, mHealth, mixed methods study, mobile phone

## Abstract

**Background:**

As survival rates for children, adolescents, and young adults with cancer improve, managing treatment-related side effects is increasingly important. Enhancing physical activity levels has been shown to be effective in reducing some of these effects. Digital interventions, such as mobile apps, offer engaging tools to promote physical activity in young populations.

**Objective:**

This article introduces the “FORTEe Get Strong” app (Nurogames GmbH) and presents a formative evaluation of its acceptability among children, adolescents, and young adults with cancer.

**Methods:**

The “FORTEe Get Strong” app was developed within the multicenter FORTEe trial. Grounded in behavior change theories, the app uses gamification to deliver a child-friendly platform promoting physical activity and health-related knowledge. An embedded mixed methods design, with qualitative insights contextualizing quantitative findings, was applied. User experience was assessed using a self-developed questionnaire combining Likert scale items (1‐5: “not at all satisfied” to “very satisfied,” 1‐3 for participants aged <8 y) and open-ended questions. Quantitative data were analyzed descriptively and inferentially, including chi-square tests for differences in app usage by age, sex, and diagnosis, and Kruskal-Wallis tests to compare app feature ratings between age groups. Free text responses underwent qualitative content analysis.

**Results:**

The app was published in May 2023; it integrates gamified features to teach health knowledge. Exercise videos address endurance, strength, flexibility, coordination, and gait. Feedback on user experience was provided by 53 patients aged 5‐19 years (mean 11.4, SD 3.8 years, 95% CI 10.4‐12.5; 27/53, 51% male). App use did not differ significantly by age groups (*χ*²_3_=5.1; *P*=.16; Cramer V=0.135), sex (*χ*²_1_=3.4; *P*=.06; *φ*=0.110), or diagnosis (*P*=.54; Cramer V=0.168). The app was generally well-received, with a mean Likert scale score of 4.14 (SD 1.04, 95% CI 3.84‐4.43). For individual components, mean scores ranged from 3.52 (SD 1.44, 95% CI 3.09‐3.95) for exercise content to 4.37 (SD 0.82, 95% CI 4.14‐4.60) for design. Kruskal-Wallis tests revealed no significant differences among age groups. Qualitative analysis identified key categories regarding overall app evaluation, specific features, target group perception, usability, and suggestions for improvement. While participants highlighted the app’s design and gamification, concerns arose regarding age-appropriateness, lack of supervision, and adaptability of exercises.

**Conclusions:**

This study provides novel insights into user experience of children, adolescents, and young adults with cancer with a gamified exercise and health education app. Unlike previous studies focusing on survivorship, this evaluation offers a comprehensive understanding of how digital tools can support patients in maintaining physical activity during intensive treatment. Apps with interactive, gamified elements may complement clinical care by providing low-threshold access to exercise and health education. The findings advance the field by identifying key facilitators and barriers to engagement. Future research should assess adherence, behavioral outcomes, and effectiveness in larger samples to inform clinical implementation.

## Introduction

Cancer is the leading cause of disease-related death among children, adolescents, and young adults. Approximately 14,000 new diagnoses were estimated in Europe in 2022 in children, adolescents, and young adults aged 0 to 19 years. Advances in treatment have significantly improved survival rates, leading to a survival rate of 81% in the mentioned age group [1]. However, this remarkable improvement in survival rates underscores the growing importance of addressing the short and long-term side effects [2,3]. Side effects can include fatigue, muscle weakness, reduced mobility, and long-term effects, such as cardiovascular diseases and metabolic disorders [2,3]. Exercise programs may offer an effective approach to addressing these issues [4]. Various studies demonstrate that exercise interventions can enhance physical aspects, such as muscle strength [5-7] and endurance capacity [5,7], as well as psychosocial outcomes, such as cancer-related fatigue [5,8] and quality of life [9,10]. Given the considerable side effects associated with the diagnosis and treatment of cancer, there is an urgent need for innovative, motivational, and age-appropriate interventions to support young patients in their physical activity and to counteract the observed side effects [11].

The increasing integration of digitalization and mobile technologies into everyday life has profoundly accelerated the use of digital tools in various domains of health promotion, including encouraging physical activity [12-15]. Among young populations, digital interventions, such as mobile apps, have become an effective and engaging tool to promote health-related behaviors [16,17]. A substantial number of these so-called mobile health (mHealth) apps designed for children, adolescents, and young adults target a variety of health outcomes, most notably physical activity [16,17].

To ensure the effectiveness of these digital interventions and to keep users motivated to engage in health-related behaviors, it is crucial to understand the mechanisms and design features that drive behavioral change, particularly in young populations. In this context, Baumann et al [16] highlight the efficacy of gamified approaches in promoting physical activity and reducing sedentary behavior in children, adolescents, and young adults, providing that these interventions are supported by individualization, a robust theoretical framework, and the integration of behavior change techniques. The role of social connections, including family, peers, and the wider community, is identified as a key factor influencing the success of mHealth strategies, demonstrating the need for interventions tailored to developmental stages and specific needs to reduce physical inactivity and sedentary behavior. Complementing this, Ghosh et al [18] investigate critical app design features that increase continued engagement and behavioral impact among adolescents. These include user-friendly and aesthetically appealing interfaces, personalized goal-setting, comprehensive data tracking, and access to educational content, such as exercise recommendations and nutritional advice. Additionally, the integration of electronic health records has the potential to facilitate data sharing with health care providers, and gamification and social interaction components are known to enhance intrinsic motivation and lead to a sense of group belonging [18]. The findings emphasize the importance of theoretically grounded and thoughtfully designed mHealth interventions in achieving substantial and lasting health-related behavior improvements in young populations.

The Europe-wide, multicenter FORTEe trial (Get strong to fight childhood cancer – An exercise intervention for children and adolescents undergoing anti-cancer treatment) aims to implement exercise as a therapy and increase physical activity using digital approaches in children, adolescents, and young adults with cancer. As part of the trial, the “FORTEe Get Strong” app (Nurogames GmbH) was developed with the primary aim of engaging children, adolescents, and young adults with cancer in physical activity and secondarily to teach health-related behaviors. Details of the study design have been published previously [19].

Despite growing evidence on mHealth solutions for young populations, little is known about how children, adolescents, and young adults with cancer themselves perceive such apps and which refinements might be necessary to support long-term engagement. Addressing these gaps requires both quantitative assessments of user experience and qualitative insights into individual needs and expectations. Therefore, the objectives of this article are to (1) introduce the “FORTEe Get Strong” app and describe its design and development, and (2) present a formative mixed methods evaluation assessing the app’s acceptability in a sample of children, adolescents, and young adults with cancer. The aim is to evaluate user experience ratings quantitatively and explore user perceptions qualitatively to contextualize the quantitative findings. Finally, both strands are integrated in a formative mixed methods evaluation, with the qualitative findings providing insight into, and helping to interpret, the quantitative data.

## Methods

### App Development

#### Overview

The “FORTEe Get Strong” app represents a component of the overall FORTEe exercise intervention, facilitating the transition from supervised, hospital-based training to self-directed exercise at home. The mobile app was developed by scientific and clinical experts of the FORTEe consortium and engineered by Nurogames GmbH. Nurogames GmbH is a software development company with expertise in creating games that, in addition to their entertainment value, contribute to the investigation of specific topics and the generation of scientific knowledge. To ensure the app resonated with its target group, patient representatives were actively involved in the design process. Feedback from patient interviews regarding their preferred theme and avatar design was essential in understanding patients’ wishes and creating an appealing and relatable design. The app has been translated into 7 languages (Danish, English, French, German, Italian, Slovenian, and Spanish) for use on Android (Google LLC) and iOS (Apple Inc) devices. It is freely available for download from the App Store (Apple Inc) and Google Play Store (Google LLC), respectively.

#### Theoretical Framework

The development of the app is based on a combination of the social cognitive theory (SCT) [20] and self-determination theory (SDT) [21]. SCT explains the interaction between personal factors, environmental influences, and behaviors, emphasizing the importance of self-efficacy, an individual’s belief in his or her ability to succeed. This belief influences goals, outcome expectations, and behavioral adoption, with environmental facilitators and barriers shaping these processes. The app’s integration of evidence-based exercise and other health-related content (eg, on healthy diet) is intended to enhance self-efficacy in patients with cancer, enabling them to set meaningful goals, create actionable strategies, and develop skills to engage in regular physical activity [20,22,23]. SDT highlights the importance of fulfilling the psychological needs for autonomy, competence, and relatedness to enhance intrinsic motivation. When these needs are met, individuals experience greater autonomous motivation, leading to sustainable behavior change. For instance, gamification features (eg, a customizable avatar and interactive quizzes) may enhance relatedness and competence, while allowing patients to select and adapt activities to their preferences and individual needs may increase autonomy [21,23,24]. Behavior change techniques, like SCT and SDT, have previously been implemented as a guideline for an innovative mobile app designed for children, adolescents, and young adults with cancer in a study by Fuemmler et al [23]. While initial evidence for actual behavior change in that study was limited, the intervention demonstrated feasibility, positive user engagement, and overall favorable reception, supporting the use of theory–based strategies as a guiding framework for developing our app.

#### Data Protection

For data protection reasons, no personal data (eg, duration or frequency of app usage) is processed in relation to the use of the “FORTEe Get Strong” app due to its implementation in a clinical setting. The data attained in the app are stored exclusively on the local device. Upon uninstallation, the app is restored to its original factory settings. As such, it does not collect or process personal data, nor does it facilitate the transfer of health information, such as electronic health records.

#### App Design, Content, and Functionality

The app’s gamification is characterized by a playful aesthetic, focusing on incentivizing physical activity as a health promotion strategy and directly providing access to individually tailored exercises. The purpose of the design of the app is to raise awareness of the importance of regular physical activity during cancer treatment and to promote intrinsic motivation for continued use of the mHealth app [18,25]. In accordance with the recommendations by Ghosh et al [18], the objective was to provide simple and accessible guidelines for various exercises, thereby empowering young users to engage in independent physical activity and support self-efficacy. The exercises were developed by the Network Active-Onco-Kids and compiled in collaboration with exercise professionals from the FORTEe consortium, ensuring their adaptation to the needs of young patients with cancer. The compilation of all educational content, as well as the exercises themselves, was undertaken by a group of scientific researchers, as well as Network ActiveOncoKids and Youth Cancer Europe, and in line with preliminary German exercise guidelines in pediatric oncology [26], with the objective of providing content that is evidence-based, freely accessible, and easily comprehensible. To further support the gamification characteristics of the app, as recommended by Fuemmeler et al [23], Baumann et al [16], and Ghosh et al [18], various mini-games are integrated in the app. To enhance motivation and thereby support continued app use, patients are encouraged through a system of rewards to engage with the extensive knowledge content and to participate in the provided exercise sessions on a regular basis.

### Evaluation of the App

#### Study Design and Rationale

As part of the FORTEe trial, the aim of this article is to conduct a formative evaluation of the mHealth “FORTEe Get Strong” app and gain insights into the experiences of children, adolescents, and young adults with cancer. This study represents a subproject and thus a secondary analysis within the overall FORTEe trial. This study adhered to the Mixed Methods Reporting in Rehabilitation and Health Sciences checklist (Checklist 1) [27].

A mixed methods approach was chosen in order to provide a comprehensive evaluation of the app, by combining quantitative and qualitative data. Quantitative data, such as user numbers and structured app ratings, capture levels of adoption and satisfaction and enable comparison across participant groups. Complementary qualitative analysis of open-ended responses adds depth by explaining why users rated the app as they did, highlighting specific usability issues and perceived benefits, and suggesting improvements. The embedded design, in which qualitative insights help explain and contextualize quantitative findings, allows both strands to complement each other, increasing the interpretability of user experiences and guiding targeted app refinements. Therefore, a self-developed questionnaire was administered between May 2023 and February 2025 with patients who participated in the randomized controlled FORTEe trial (prospectively registered on March 21, 2022). A self-developed tool was used as no existing validated instrument covered the specific app features that were intended to be assessed. Participants either responded in an interview format or answered the questions independently in written form. The interviews were not recorded. As the questionnaire or interview guidance was exploratory and context-specific, no formal psychometric validation was conducted. However, the items were reviewed and refined by pediatric oncologists (n=2) and exercise experts (n=4) on criteria including clarity of wording and developmental appropriateness. In order to minimize the influence of parents or study staff, participants were encouraged to respond independently. Parents or study staff were only permitted to provide clarification when necessary. Whenever feasible, participants were seated separately from their parents to support independent responding. The English version of the questionnaire is provided as Multimedia Appendix 1.

The integration of quantitative and qualitative data, expert review of the questionnaire or interview guidance items, and the standardized administration procedures used were intended to ensure methodological rigor and enhance the credibility and trustworthiness of the findings.

#### Participants and Recruitment

Participants were recruited within the multicenter FORTEe trial [19]. Patients were eligible if they had been diagnosed with cancer according to the International Classification of Childhood Cancer, Third edition, were between 4 and 21 years of age, and received chemotherapy and/or radiotherapy at one of the FORTEe trial sites. Participants for the app evaluation were purposively sampled from the FORTEe trial cohort, as they were the intended users of the “FORTEe Get Strong” app and could provide relevant insights into its acceptability and usability. The questionnaire has been performed at all participating recruitment centers of the FORTEe trial—University Medical Center of the Johannes Gutenberg-University Mainz, Childhood Cancer Center, Mainz (Germany); Fondazione Monza e Brianza per Il Bambino e La Sua Mamma, Monza (Italy); School of Sport, Nutrition and Allied Health Professions, Oxford Brookes University, Oxford (United Kingdom); Heidelberg University Hospital and National Center for Tumor Diseases, a partnership between DKFZ and University Medical Center Heidelberg, Germany, Department of Medical Oncology, Working Group Exercise Oncology, Heidelberg (Germany); Center de Lutte Contre le Cancer Léon Bérard, Lyon (France); Department of Pediatrics and Adolescent Medicine, Copenhagen University Hospital, Rigshospitalet, Copenhagen (Denmark); Universidad Europea de Madrid, Faculty of Medicine, Health and Sports, Department of Sport Sciences, Madrid (Spain); Research Institute of the Hospital 12 de Octubre (‘imas12’), Madrid (Spain); University Hospital Essen, West German Cancer Center, Essen (Germany); Fondazione IRCCS Istituto Nazionale dei Tumori, Pediatric Oncology Unit, Milan (Italy); and University Medical Center Ljubljana, Ljubljana (Slovenia) in cooperation with Forma 3D Ltd, Ljubljana (Slovenia).

### Ethical Considerations

The FORTEe study protocol and all related documents were approved by the Ethics Committee of the Medical Chamber of Rhineland-Palatinate (application 2021‐15904 on August 4, 2021) as well as the local ethics committees of all FORTEe trial sites and reviewed by the local data protection officer. Written informed consent was obtained from all participants, or from their legal guardians in the case of minors. Where possible, documented assent was collected from children aged 6 years and older. All participant data were pseudonymized before analysis. Identifying information was accessible only to authorized study personnel, safeguarding privacy and confidentiality. Participants in this evaluation did not receive any financial or material compensation. All procedures adhered to the principles outlined in the Declaration of Helsinki. Neither the paper nor the supplementary materials contain images of the study participants. Any individuals depicted are nonparticipant staff members, and it is not possible to identify individual study participants.

### Method of Assessment

#### Overview

The questionnaire was designed to gather participants’ perspectives regarding their use of technology. With regard to the “FORTEe Get Strong” app, it included questions about user satisfaction with the app as a whole, the design of the app, the FORTEe avatar, the range of exercises, the FORTEe quiz, the knowledge content included and the app’s user-friendliness. The interviews were conducted by local exercise professionals with experience in pediatric oncology in their native language and translated into English language for further analysis. Their previous understanding of childhood cancer treatment and exercise interventions informed probing questions, while efforts were made to minimize bias by following standardized guidelines and allowing independent participant responses. To further support comprehension, interviewers followed standardized guidelines that allowed them to adapt the wording of items to the developmental level of the participants while preserving the original meaning.

#### Assessment of Quantitative Data

The quantitative component of the evaluation consisted of questions presented on a 5-point or 3-point Likert scale, depending on the age of the participant. The response options ranged from “not at all satisfied” to “very satisfied” and were illustrated by child-friendly emojis. The 3-point scale, developed for use of participants under the age of 8 years, included only the response options 1 (“not at all”), 3 (“somewhat”), and 5 (“very much”). The 5-point scale, which includes the additional options 2 (“hardly”) and 4 (“quite a lot”), is used by participants aged 8 years and older. This age-differentiated scaling approach was chosen because it provides younger children with fewer, more distinguishable response categories [28-31].

#### Assessment of Qualitative Data

Furthermore, participants were invited to provide additional comments regarding their thoughts on the “FORTEe Get Strong” app in an open-ended format, allowing for qualitative analysis of their individual experiences and perceptions.

### Study Size

This formative evaluation was embedded within the main FORTEe randomized controlled trial. No separate power calculation was performed for this app evaluation. The overall FORTEe trial included an a priori sample size calculation for the primary outcome (cancer-related fatigue), targeting 450 participants [19]. The sample for this evaluation comprises FORTEe participants who took part in the app evaluation during the data collection period.

### Data Analysis

#### Overview

The ages of the participants were recorded as continuous variables and subsequently grouped into 4 predefined categories to enable group-based comparisons. According to Sawyer et al [32], chronological age boundaries are both arbitrary and context-dependent, varying according to cognitive, social, and cultural factors. For the FORTEe trial and therefore for the “FORTEe Get Strong” app, age groups were defined based on cognitive development, literacy, and school-related competencies, in order to ensure age-appropriate interaction and consent procedures. The age groups are (1) 4‐5 years (preschoolers), (2) 6‐11 years (school children), (3) 12‐16 years (adolescents), and (4) 17‐21 years (young adults) [19].

#### Analysis of Quantitative Data

For quantitative analyses, descriptive continuous variables are expressed as mean values with SDs and 95% CIs, while categorical variables are presented as the actual number of participants, accompanied by the corresponding percentage. For analysis and visualization, responses from the 3-point scale (used for participants younger than 8 y) and the 5-point scale (used for participants aged 8 y and older) were combined, with equivalent points being interpreted as the same level of satisfaction. A chi-square test was performed to investigate whether app usage differed across the defined age groups, sex, or diagnosis. If the assumption that the expected frequency in at least 20% of cells was less than 5 was violated, a Fisher-Freeman-Halton exact test was performed instead. A Kruskal-Wallis test was used to investigate possible differences in the evaluation of individual app features by age group. The significance level was set at 5%.

#### Analysis of Qualitative Data

Complementing the quantitative evaluation, qualitative content analysis, as outlined by Mayring [33], was used to facilitate a systematic examination of the free-text responses. A pragmatic interpretive approach guided the analysis, focusing on understanding participants’ experiences and perceptions of the app in context. The objective was to categorize the responses and record the frequency of occurrence. A repeated review of all responses was conducted to identify key themes, and categories were formed inductively. The process of open coding was used to extract the recurring themes and group them into overarching categories. This was performed independently by 2 researchers (MK and LW), and discrepancies were resolved through discussion. The categories identified reflect the principal topics addressed in the free text responses. Categories were developed inductively, ensuring consistent interpretation across the dataset. A quantitative analysis was then conducted, counting the frequency with which each category appeared in the responses. This provides an initial approximation of the relative importance of the different topics.

#### Mixed Methods Integration

Finally, both data strands were integrated using an embedded mixed methods design, in which quantitative data provided an overview of adoption and satisfaction, and qualitative insights helped explain and contextualize the quantitative findings. This approach allows the complementary strengths of both methods to reinforce the conclusions, while acknowledging their respective limitations. The integrated analysis enables a richer interpretation of the results and informs targeted app refinement and implementation strategies, illustrating the added value of using a mixed methods design.

The descriptive statistical analyses were conducted using IBM SPSS (version 27), while qualitative content analysis was carried out using Microsoft Excel (version 2016, 16.0.5474.1002). The graphs were created with R statistical software (RStudio; version 2024.09.1).

## Results

### The App Development

#### Overview

**Figure 1.**
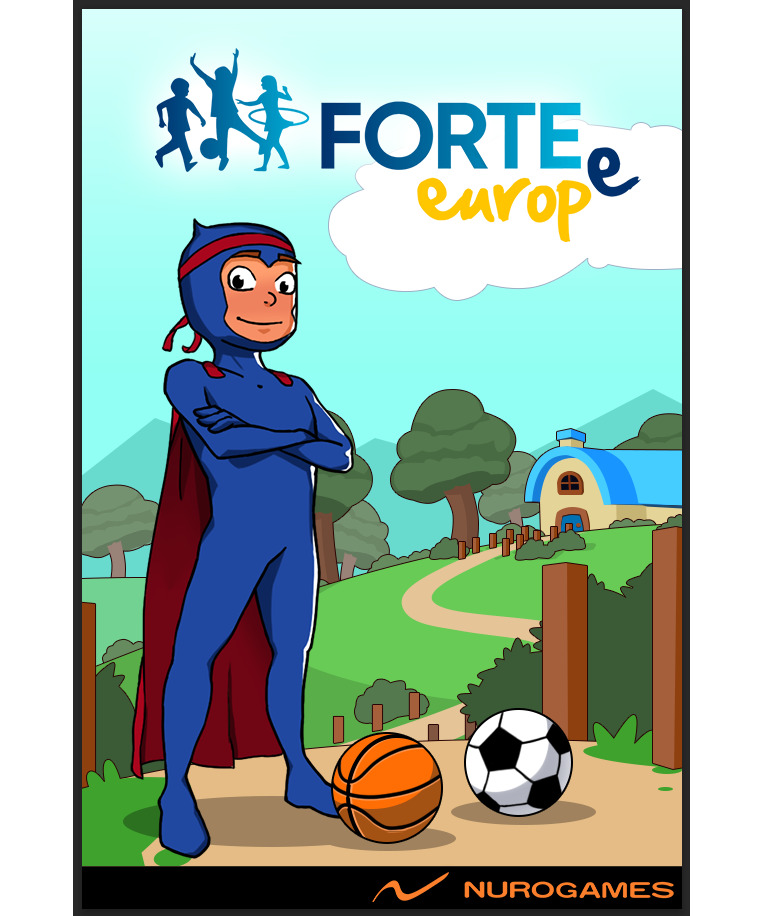
The “FORTEe Get Strong” app.

The “FORTEe Get Strong” app (Figure 1) was published in May 2023 for use on Android (Google LLC) and iOS (Apple Inc) devices and is now freely available for download from the App Store (Apple Inc) and Google Play Store (Google LLC). Minor technical updates were made to the “FORTEe Get Strong” app during the evaluation period. These included correcting language inconsistencies, optimizing in-app texts, and resolving minor software bugs. The following versions were released, used, and evaluated by participants—version 1.0.1, version 1.1.1, and version 1.1.2. As all versions were functionally identical in terms of content and user interface, they were evaluated collectively.

#### App Design, Content, and Functionality

**Figure 2.**
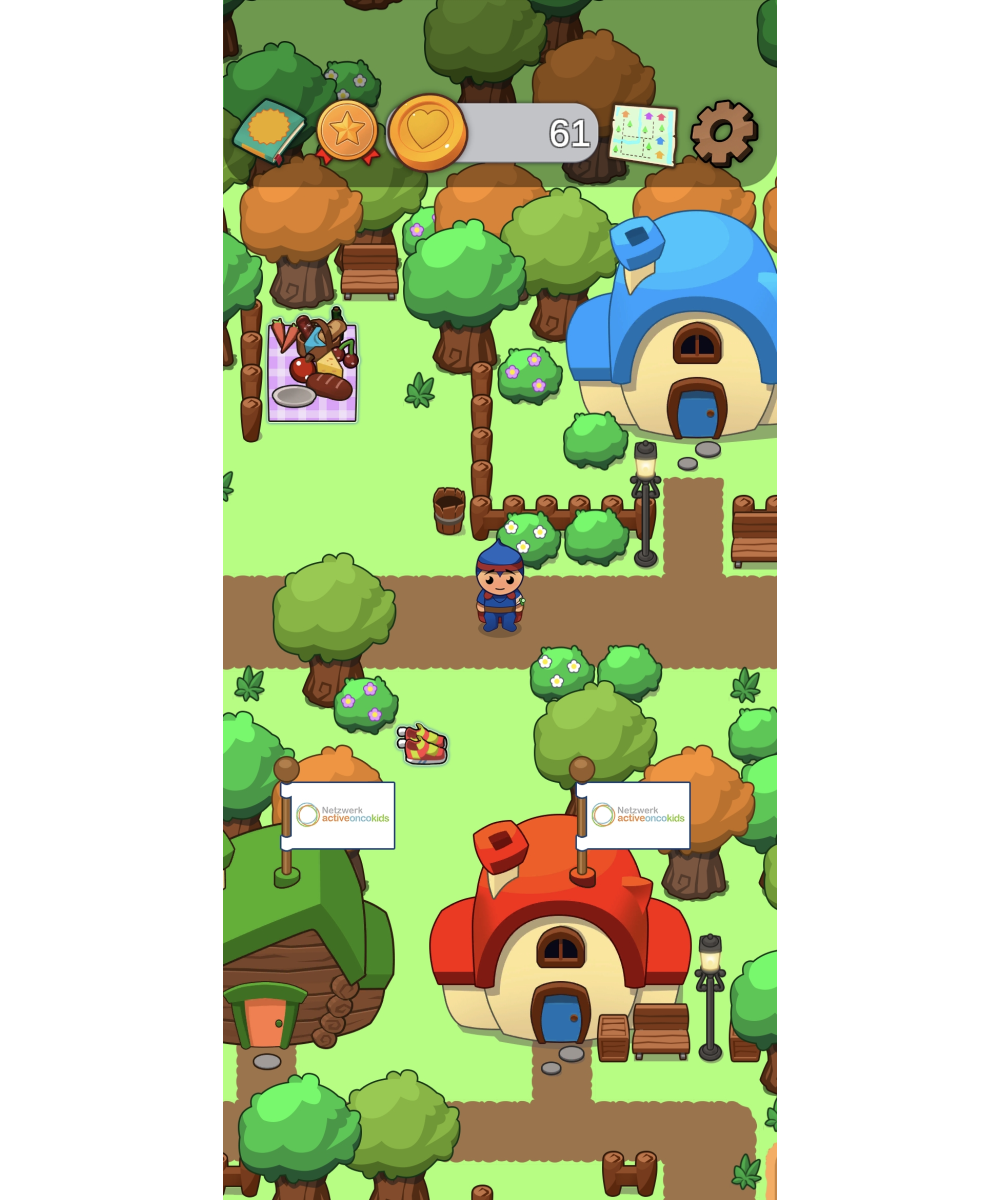
The FORTEe avatar can move around the FORTEe village, interact with the characters in the village, and can explore various health content.

The child-friendly app enables users to access a virtual environment, the FORTEe village (Figure 2). Within this virtual space, users can navigate their own avatar through a bright and colorful world containing educational content and entertaining facts focusing on physical activity and providing a range of exercises to improve different physical fitness components. The avatar includes features such as an infusion needle, bandage, alopecia, and a superhero cape, symbolizing the experiences of children and adolescents with cancer and fostering a sense of identification and empowerment when using the app.

The interaction with child-friendly characters living in the FORTEe village enables users to explore relevant knowledge regarding physical activity during cancer treatment, as well as general information on a healthy lifestyle in a playful way. The educational content is tailored for children, adolescents, and young adults with cancer and provides guidance on topics such as safe exercise during intensive treatment, changes in muscle strength, fall prevention, and healthy nutrition and an active lifestyle. This information is reinforced through quizzes and mini-games. Additionally, by visiting the FORTEe house or the numerous market stalls located in the village center, users can gather insights into the background of the app—the randomized controlled FORTEe trial.

**Figure 3.**
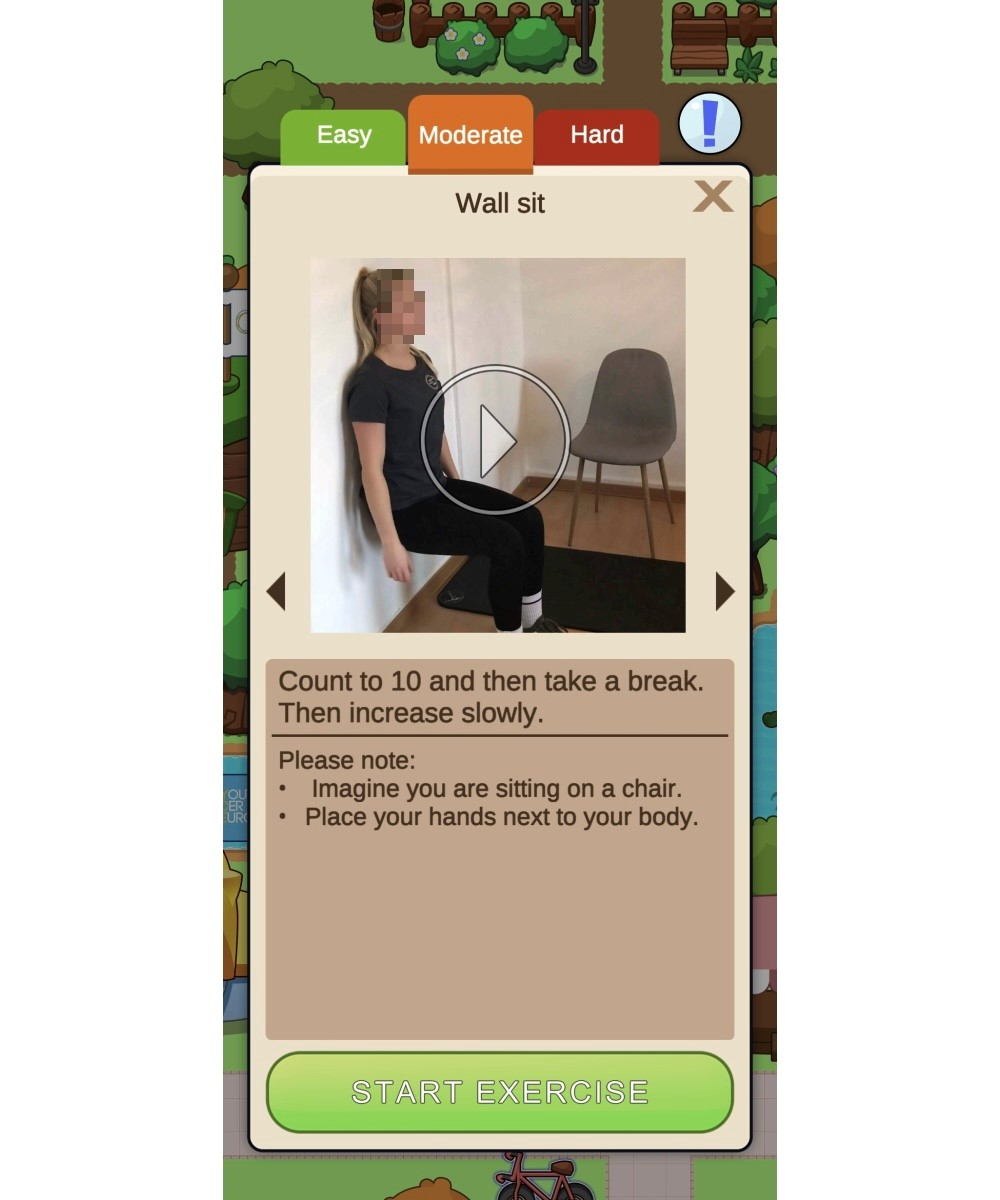
The app offers different exercises for different functions, eg, strength exercises (such as “wall sitting” with video instructions) and different levels of difficulty.

The FORTEe village consists of a series of exercise houses, each designed to target physical fitness components, including endurance, strength, flexibility, coordination, balance, and gait. The exercises are presented as exercise videos, accompanied by instructions on correct execution and number of repetitions, encouraging users to imitate and perform the demonstrated exercises themselves. The exercises are presented with a range of levels of difficulty, thus allowing for adaptation to the user’s physical ability and current health status (Figure 3). Many exercises can be performed while sitting or lying down, enabling participation by patients with reduced mobility or an increased risk of falling.

**Figure 4.**
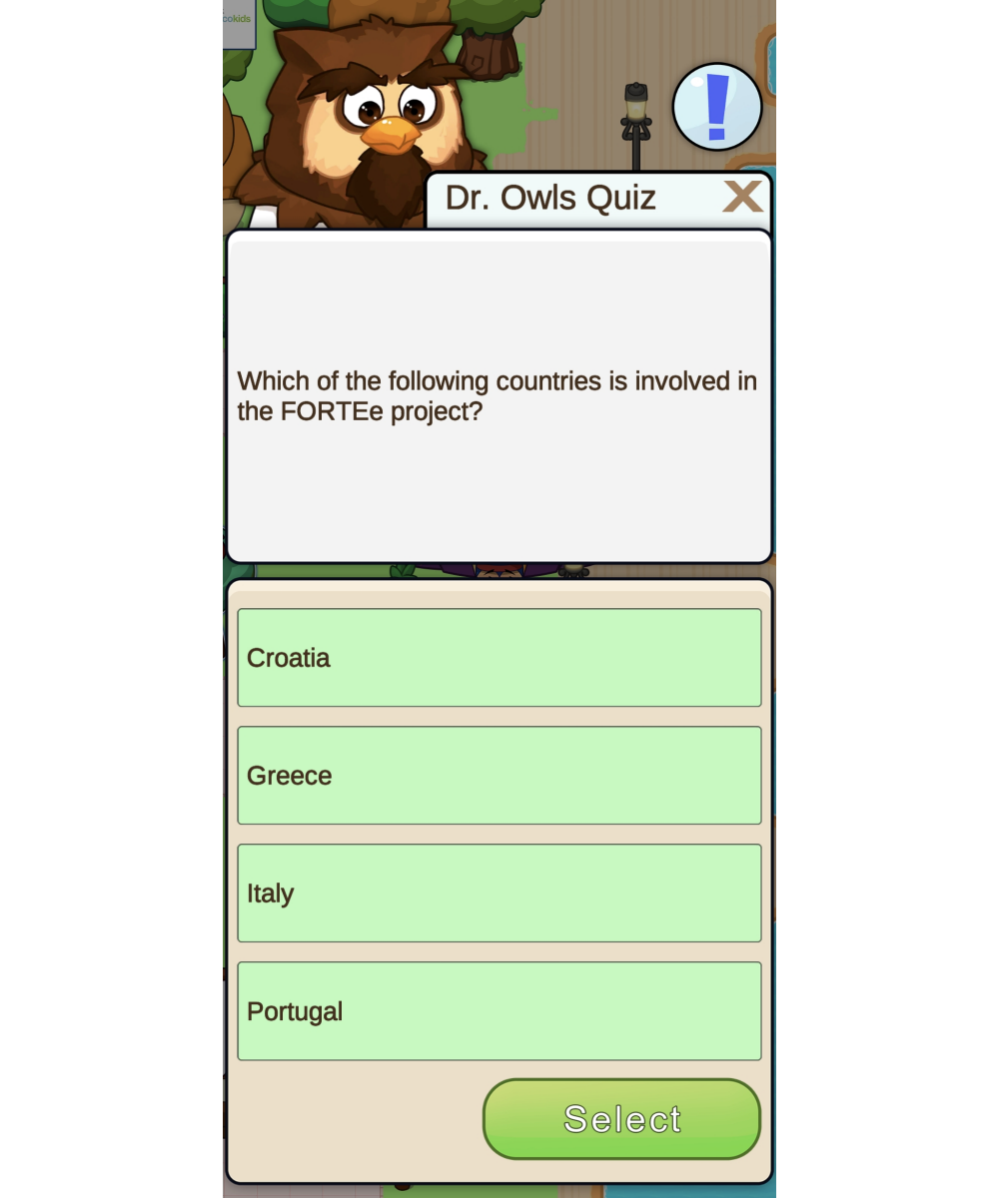
Dr Owls quiz is a fun way to learn about physical activity, exercise, and other health topics, as well as information about the FORTEe trial.

**Figure 5.**
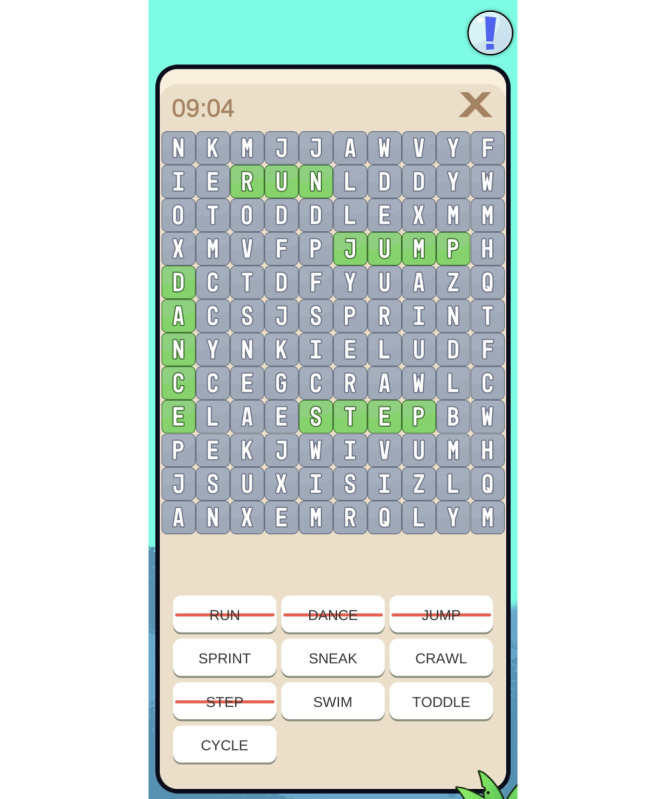
Other mini-games included in the app are a crossword puzzle, a memory game, and a nutrition puzzle.

The app also provides various mini-games, including a FORTEe quiz (Figure 4), memory games, and others (Figures5  6). Focusing primarily on physical activity, but also on a healthy diet, the mini-games are a fun way to introduce health-related topics and provide an insight into the FORTEe trial and the European partners involved in the study. By integrating the smartphone’s gyroscope, some interactive exercises within the app require users to move or shake the device in specific directions to complete simple in-game challenges. This feature transforms the smartphone into an active interface that requires physical motion, thereby playfully encouraging body movement. Finally, the app contains a “Parents” section that provides guidance on how to support their child in exercising safely during treatment. This section covers topics such as infection risk, postoperative activity, exercising with infusion stands, fall prevention, and information on peripheral neuropathy and bone metastases.

**Figure 6.**
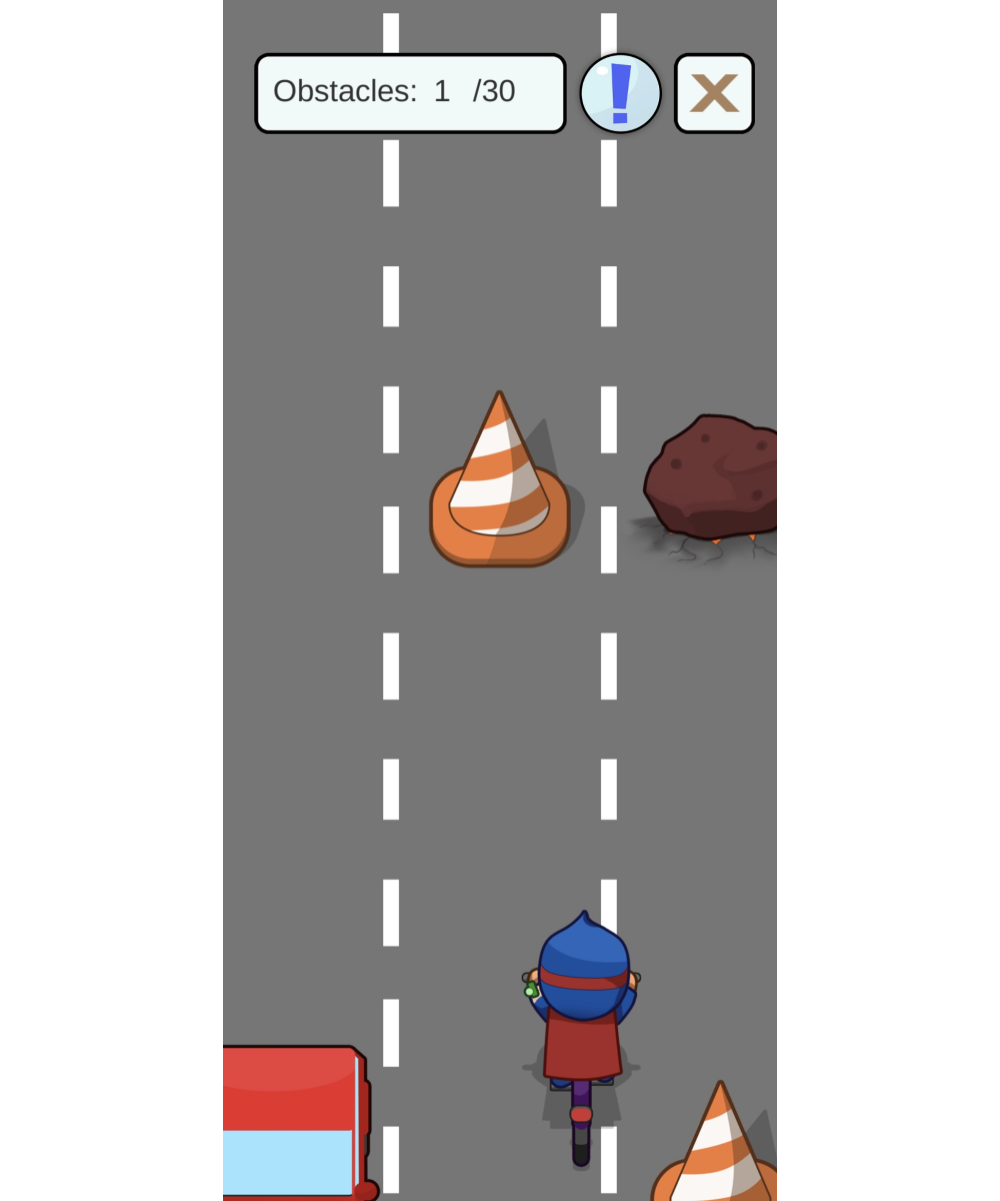
Some mini-games also offer the option to use the gyroscope of the device to directly encourage movement. This allows users to engage in activities such as running, cycling, or surfing with their avatar, overcoming various obstacles, helping the avatar in picking apples from a tree, or guiding a frisbee through the air by moving, tilting, or shaking the phone.

**Figure 7.**
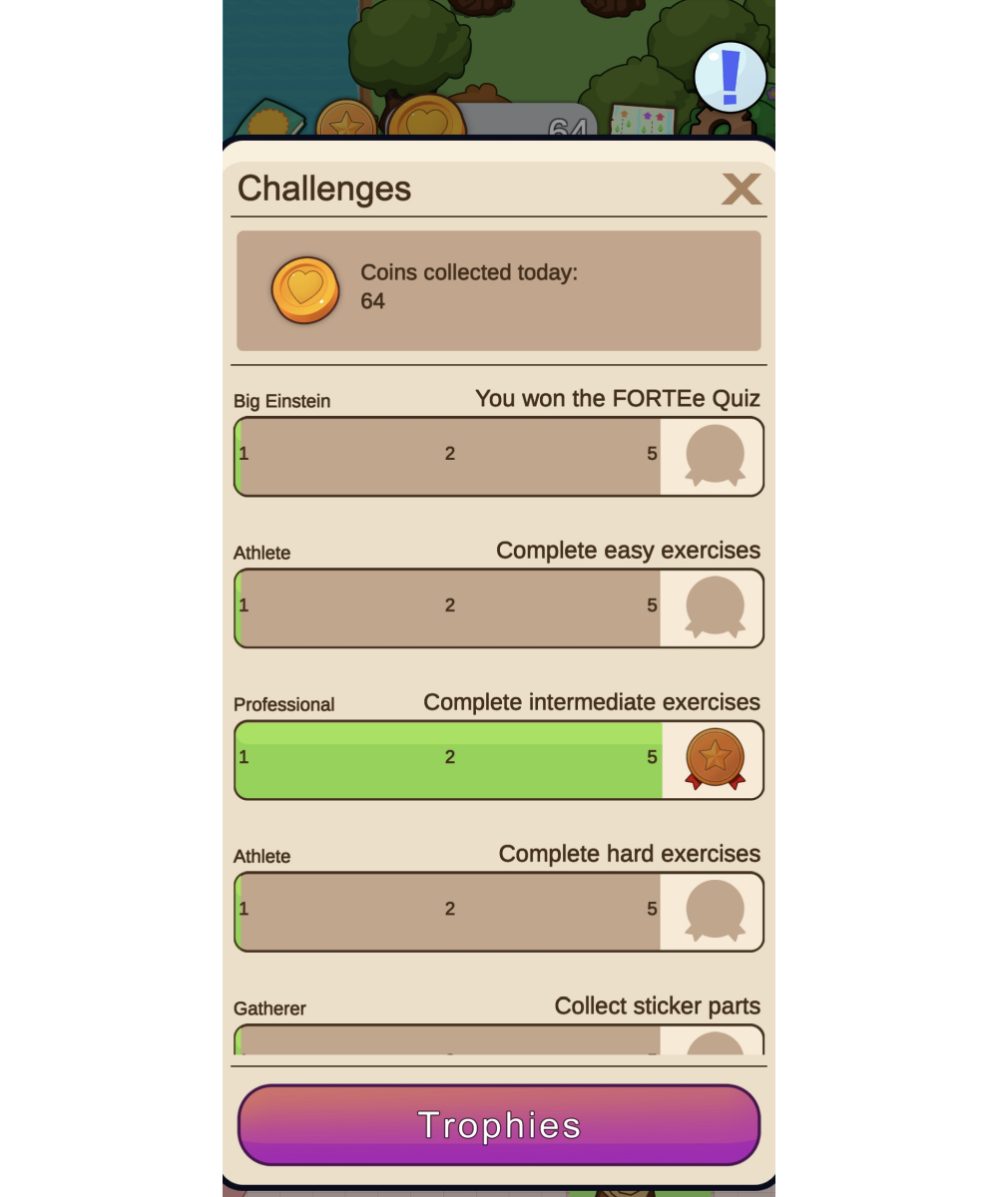
Various FORTEe challenges with the intention to motivate users to interact with the app and engage with the rich health content and the reward system.

Patients are encouraged to use the app through a reward system. In addition to unlocking new content, patients will be rewarded with FORTEe coins, which can be used to personalize their avatar and customize their avatar’s house. Various challenges also motivate exploration of the app and performance of exercise sessions. Completing challenges is rewarded with virtual medals and trophies that can be viewed in the trophy collection, to further enhance the extrinsic motivation of the users (Figure 7).

In the context of supervised exercise sessions at the FORTEe treatment centers, exercise professionals have the option of providing patients with a code as part of the rewarding system. These codes can be used in the app to unlock puzzle pieces, which, when assembled, yield engaging exercise-related images. This feature aims to enhance patient engagement, thereby promoting active participation in the supervised sessions, while making the experience more appealing and gratifying.

### Evaluation of the App

#### Overview

**Figure 8.**
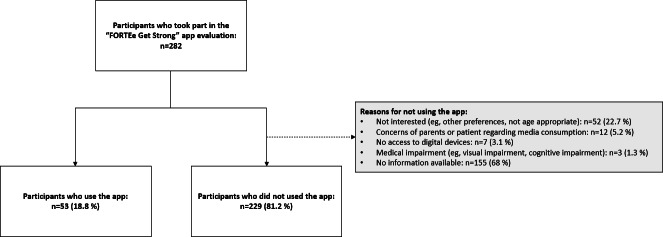
Participant flow in the evaluation of the “FORTEe Get Strong” app, showing user status and reasons for nonuse.

A total of 282 participants took part in the evaluation regarding the “FORTEe Get Strong” app; thereof, 229 participants did not use the app. The reasons for not using the app are presented in Figure 8.

A total of 53 participants indicated having used the app and further provided detailed feedback. The number of responses varies between items because not all participants answered every question. Table 1 presents the characteristics of all participants who took part in the evaluation, as well as of those who used the app.

Group comparison analyses revealed no significant differences in app usage across age groups (*χ*²_3_=5.1; *P*=.16; Cramer V=0.135), sex (*χ*²_1_=3.4; *P*=.06; *φ*=0.110), or diagnosis (*P*=.54; Cramer V=0.168).

**Table 1.** Characteristics of all evaluated participants and of those who used the app^a^.

	All evaluated participants (n=282)	App users (n=53)
Age (y)		
Mean (SD)	11.8 (4.3)	11.4 (3.8)
Median (Range)	12 (4‐21)	11 (5‐19)
Age group, n (%)		
Pre-schoolers (4‐5 y)	25 (9)	2 (4)
School children (6‐11 y)	104 (37)	26 (49)
Adolescents (12‐16 y)	109 (39)	18 (34)
Young adults (17‐21 y)	44 (16)	7 (13)
Sex, n (%)		
Female	112 (40)	26 (49)
Male	170 (60)	27 (51)
Diagnosis, n (%)		
Leukemias	106 (38)	21 (40)
Lymphomas	59 (21)	12 (23)
CNS^b^ tumors	25 (9)	7 (13)
Neuroblastoma	7 (2)	1 (2)
Renal tumors	4 (1)	1 (2)
Malignant bone tumors	40 (14)	7 (13)
Soft tissue tumors	16 (6)	1 (2)
Germ cell tumors	15 (5)	1 (2)
Others	10 (4)	2 (4)

a Percentages may not total 100% due to rounding.

b CNS: central nervous system.

#### Patient Experience of the “FORTEe Get Strong” App

##### Results of the Quantitative App Evaluation

Overall, the “FORTEe Get Strong” app was well-received by the patients surveyed, receiving an average rating of 4.14 (SD 1.04) points out of 5 points. A clear majority of 71% (36/51) of participants gave the app an overall positive rating (“I liked the app quite a lot” or “I liked the app very much”), while only 6% (3/51) gave a negative rating (“I hardly liked the app” or “I liked the app not at all”). In particular, the design (mean 4.37, SD 0.82 points; 42/51, 82% positive ratings; 1/51, 2% negative ratings), the user-friendliness (mean 4.14, SD 1.11 points; 37/50, 74% positive ratings; 4/50, 8% negative ratings), and the avatar (mean 4.14, SD 1.10 points; 36/51, 71% positive ratings; 3/51, 6% negative ratings) were well-received. The quiz received an average rating of 3.89 (SD 1.07) points, with 61% (23/38) positive and 8% (3/38) negative feedback.

Opinions on the exercises and the informational content were more varied. Although more than half of the participants rated these features positively, nearly a quarter provided negative ratings. This represents a notably higher proportion of negative feedback than for the other app components. The average rating of the exercises was 3.52 (SD 1.44) points, with 52% (24/46) of participants rating the exercises positively and 22% (10/46) negatively. The informational content received an average rating of 3.64 (SD 1.48) points, with 60% (28/47) of participants giving positive ratings and 23% (11/47) giving negative ratings. Kruskal-Wallis tests revealed no significant differences in the evaluations of the various specific app features examined among age groups (Table 2).

**Figure 9.**
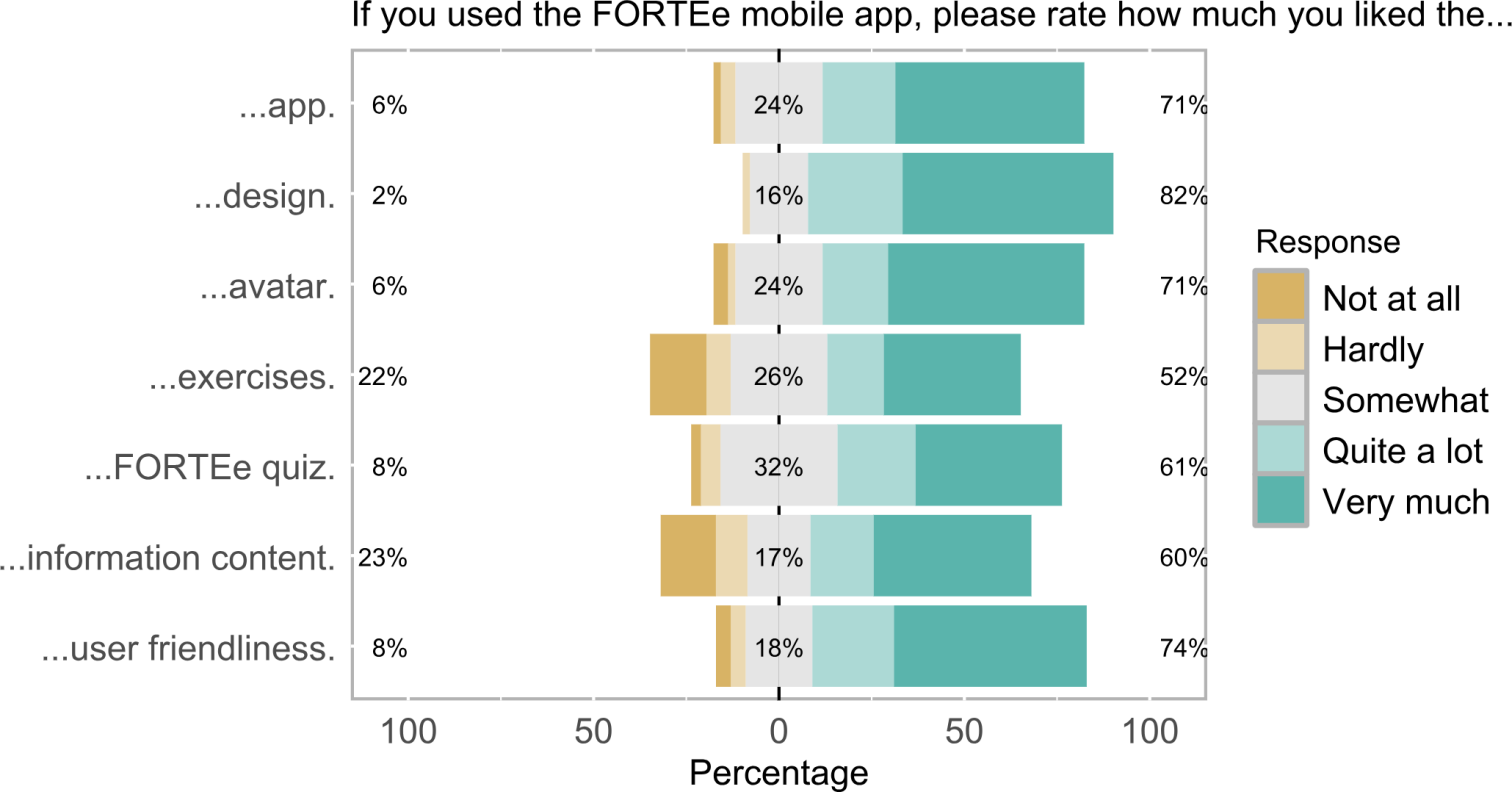
Results of the app evaluation. Participants rated various features of the “FORTEe Get Strong” app including the app in general, its design, the avatar, the exercises, the FORTEe quiz, the informational content as well as the user-friendliness rated on a scale from “Not at all” to “Very much.” The percentages at the bottom show the distribution of ratings relative to the central reference point (0%), with negative values extending to the left and positive values extending to the right. The percentage of negative ratings (“I liked the app not at all” or “I hardly liked the app”) is shown on the left (yellow), the percentage of neutral ratings (“I liked the app somewhat”) is shown in the middle (gray), and the percentage of positive ratings (“I liked the app quite a lot” or “I liked the app very much”) is shown on the right (green).

The quantitative analysis demonstrated that the app was generally well-received, particularly in terms of its design, user-friendliness, and avatar. However, the ratings for exercises and informational content exhibited greater variability, with more than half of the participants providing positive ratings but nearly a quarter expressing negative feedback. Table 2 and Figure 9 present a summary of the quantitative rating of the app components.

**Table 2.** Results of the quantitative app evaluation, including comparisons across age groups^a^^,^^b^.

If you used the FORTEe mobile app, please rate how much you liked the…	n	Mean (SD)	95% CI	H (*df*)	*P* value
…app.	51	4.14 (1.04)	3.84‐4.43	1.66 (3)	.65
…design.	51	4.37 (0.82)	4.14‐4.60	1.42 (3)	.70
…avatar.	51	4.14 (1.10)	3.83‐4.45	1.42 (3)	.70
…exercises.	46	3.52 (1.44)	3.09‐3.95	1.81 (3)	.61
…FORTEe quiz.	38	3.89 (1.09)	3.54‐4.25	3.27 (3)	.35
…informational content.	47	3.64 (1.48)	3.20‐4.07	2.56 (3)	.46
…user friendliness.	50	4.14 (1.11)	3.83‐4.45	0.99 (3)	.81

a Values are presented as mean (SD) and 95% CIs.

b Group differences between age groups were analyzed using Kruskal-Wallis tests.

##### Results of the Qualitative App Evaluation

The qualitative analysis of the free text fields led to the identification of several central categories, such as (1) overall app evaluation, (2) evaluation of specific features (design, exercise content or app as a training tool, mini games, and informational content), (3) target group perception (age relevance), (4) app usability, and (5) suggestions for improvement, which summarize the experiences and opinions of the participants about the app. Thematic saturation was deemed achieved because responses repeatedly reflected the same set of themes, and no new categories emerged in the later stages of analysis. The main results are presented further in this study and in Table 3.

In total, 12 respondents provided additional positive feedback on the app in general, describing it as “very cool and fun” and said that the “app is nice during hospitalization,” highlighting the opportunity to “use it offline.” Moreover, 5 participants gave negative feedback, stating that the app had become uninteresting over time. With regard to the exercise content, patients (n=2) indicated that the exercises accessible via the app were beneficial. However, 5 patients stated that the range of exercises was less sophisticated than those offered in supervised settings and that they preferred to engage in exercise sessions with an exercise professional. In addition, 5 patients provided feedback on the design of the app, with 4 patients specifically mentioning the design of the avatar. However, 1 patient gave negative feedback on the design of the app. Furthermore, 2 patients provided comments on the mini-games, offering exclusively positive feedback. The feedback on the informational content of the app was mixed. One participant commented that the information about other centers was “nice to learn about,” while 3 described the information as “too long” or stated that it was “more suitable for adults.” Of the total, 7 patients provided feedback on the suitability of the app for their age group. The most common feedback from adolescents and young adults was that the app was perceived as more useful and engaging for younger children, or that the patients themselves felt that they were too old for the app. Moreover, 2 patients provided feedback indicating that the game control did not function as effectively as desired, and that “the app is not very intuitive to use.” A total of 5 patients provided feedback on potential improvements to the app, giving both design-related suggestions and requests for additional features. Such suggestions included the addition of a chess game or a virtual fitness studio.

**Table 3.** Qualitative evaluation of the design and content of the “FORTEe Get Strong” app with main categories and subcategories to assess patient perspectives. Of the 53 patients included in the evaluation, 49 provided responses that could be assigned to the defined subcategories. Multiple responses per patient were possible, while some patients did not provide any feedback.

Main category and subcategory	Example quotes	Patients, n
Overall App evaluation		
General		
Positive	“The app is very cool and fun.” “The app is nice during hospitalization.” “Especially during radios (no internet) I was able to use it offline”	12
Negative	“It feels like it’s not finished yet.” “The app got boring after a while.”	5
Evaluation of specific features		
Design		
Positive	“I liked the avatar.” “The avatar is cute.”	4
Negative	“I didn’t like the design of the app.”	1
Exercise content or app as a training tool		
Positive	“I liked the exercises/workouts.” “The exercises are helpful.”	2
Negative	“The app’s exercise offer is less sophisticated compared to other (supervised and supported) exercise offers.” “It is much more interesting to exercise with exercise professionals in real.” “The exercises are too easy.”	5
Mini games		
Positive	“I like the games, they are funny.” “The quizzes are nice.”	2
Informational content		
Positive	“It is nice to learn about other centers.”	1
Negative	“The information content of the app is more suitable for adults.” “The information about the other centers is too long.”	3
Target group perception		
Age relevance	“I am 20 years old and it is not funny for me. But nice idea for the youngest.” “Only a very small age group is suitable for the app.” “I like the games; they are funny, but more appropriate for younger children.”	7
App usability	“The avatar moves too slowly. The game control doesn’t work well.” “The app is not very intuitive to use”	2
Suggestions for improvement	“I would like the game design to be different, for example the first-person view of the player from the inside (more real).” “You must add a chess game.”	5

##### Mixed Methods Integration

The integration of quantitative and qualitative data provided a comprehensive understanding of users’ experiences with the “FORTEe Get Strong” app. Quantitative findings indicated an overall positive evaluation. Qualitative findings from open-ended responses supported and expanded these results. Participants frequently described the app as “fun,” “cool,” and particularly useful during hospitalization, confirming the generally positive quantitative ratings. However, several users expressed different concerns, providing context for the variability observed in the quantitative ratings of some features. Taken together, the integration of quantitative and qualitative data revealed converging findings regarding the app’s strong usability and appeal, while qualitative insights further clarified user preferences and contextualized the quantitative variability observed for certain app features.

## Discussion

### Summary

This mixed methods study aimed to (1) introduce and describe the development of the “FORTEe Get Strong” app, and (2) evaluate its usability, acceptability, and user experience among children, adolescents, and young adults with cancer participating in the multicenter FORTEe trial. The “FORTEe Get Strong” app was developed to promote physical activity and health awareness among children and adolescents with cancer, by including behavior change theory and gamification principles to foster motivation, engagement, and long-term participation in physical activity. By integrating quantitative patterns with qualitative explanations, the embedded mixed methods approach was used to provide a comprehensive understanding of user experience. The evaluation included data from 53 children, adolescents, and young adults with cancer who used the app within the FORTEe trial. The quantitative assessment revealed that the app was generally well-received, with high ratings for the app in general, the design, the user-friendliness, and the avatar. Ratings for exercise content and informational materials were more variable. Group comparison analyses revealed no significant differences in app usage according to age group, sex, or diagnosis. Similarly, there were no significant differences in evaluations of certain app functions among the 4 age groups. Qualitative content analysis of open-ended responses produced several themes, such as “overall app evaluation,” “evaluation of specific features” (including “design,” “exercise content or app as a training tool,” “mini games,” and “informational content”), and “target group perception” dealing with “age appropriateness,” “app usability,” and “suggestions for improvement.” Participants described the app as “fun” and “cool,” and found it particularly useful during hospitalization. However, feedback also highlighted limitations, including a perceived lack of supervision, limited adaptability of exercises, and concerns about age appropriateness. Overall, the findings of this mixed methods evaluation indicate that the app is usable, engaging, and well-received by children, adolescents, and young adults with cancer, while also highlighting clear directions for future refinement to enhance its motivational and educational value.

### Synthesis With Existing Knowledge

#### The App Development: Theoretical Framework

From a theoretical point of view, the “FORTEe Get Strong” app integrates key elements of SCT [20], particularly observational learning and self-efficacy. Exercise videos demonstrating the correct performance of exercises allow users to learn by watching, while adjustable difficulty levels build confidence in their abilities. The app further applies SCT principles through reinforcement via gamified rewards, outcome expectations conveyed through educational content, and reciprocal determinism by providing an adaptive, feedback-driven environment that promotes autonomy, long-term engagement, and long-term behavior change [20]. However, a critical aspect of Bandura’s framework [20], social modeling through peer or caregiver interaction [22], is lacking, primarily due to data protection restrictions. This hinders the app’s ability to promote social reinforcement, a key element in facilitating behavioral change [20,22]. The app’s alignment with SDT [21] is evident in its promotion of autonomy and competence. Users are given autonomy to choose their activities, customize their avatars, and set their own pace, giving them a sense of control and personal achievement. The structured and progressive nature of the exercise options, reinforced by clear instructions and a reward system, serves to enhance competence. However, the limited adaptability of the exercises in the app (only 3 difficulty levels) may restrict opportunities for individualized progression and thus only partially address the SDT need for competence [21,24]. Similarly, the lack of continuous supervision and real-time social interaction limits the extent to which the app can support the SDT need for relatedness [21,24]. While features that connect the supervised training as part of the FORTEe exercise intervention with digital rewards received in the app provide some connection, the lack of real-time social interaction with peers or caregivers limits the sense of connection and community, which is critical to developing long-term intrinsic motivation [21,24]. In comparison with the recommendations outlined by Ghosh et al [18] for mHealth apps targeting adolescents, the “FORTEe Get Strong” app effectively incorporates gamification, evidence-based educational content, and personalized exercise programs. However, it does not adequately address the need for social connectedness and individualized progression, which are highly valued by young people, caregivers, and health care providers. Moreover, the absence of ongoing supervision further limits opportunities for personalized feedback and adaptive guidance, both of which could strengthen users’ sense of competence and relatedness. The app’s limitations regarding opportunities for real-time interaction, personalized feedback, and peer engagement are primarily due to strict data protection regulations and the multicenter study design. This trade-off exemplifies the tension between maintaining high standards of data protection and implementing features that could potentially support behavioral change. While the app successfully upholds high data protection standards, these constraints, stemming from the requirements of a multicenter study and strict privacy regulations, made real-time supervision and more individualized adaptation infeasible. Future interventions could explore the integration of privacy-preserving methods for social modeling, adaptive feedback, and individualized progression to better address all 3 SDT needs of autonomy, competence, and relatedness [21,24].

#### Evaluation of the App by Patient Representatives

Of the 282 participants who completed the survey, 53 reported using the “FORTEe Get Strong” app and provided detailed feedback. While the overall number of app users was relatively small, understanding the reasons for nonuse provides important context. A considerable number of patients indicated disinterest in the app as a digital tool, preferring supervised exercise sessions at the hospital or independent training at home. This highlights the challenge of addressing diverse user needs and preferences within a single digital health solution. Some participants also perceived the app as not age-appropriate. However, this perception was not entirely reflected in the usage data. Although 37% (104/282) of all respondents were school-aged children between 6 and 11 years old, nearly half of the app users (26/53, 49%) were in this age group. Descriptively, app usage appeared higher among younger participants; however, the chi-square test revealed no significant difference by age. Similarly, despite 60% (170/282) of all respondents being male, app users were evenly distributed between females and males, and no significant difference in app usage regarding sex was found. Compared with previous studies linking older age and female sex to higher engagement with mHealth apps among adolescents [34,35], our data revealed no significant effects of age or sex. Nevertheless, the perception of the app as being more suitable for younger children may have reduced uptake among older adolescents, despite the absence of statistically significant age effects. Some parents also expressed concerns about increased media consumption resulting in their children not using the app. Those concerns are understandable, as excessive media consumption in children and adolescents can be associated with negative effects on sleep, attention, and learning, as well as higher incidence of obesity and depression [36]. In order to overcome these challenges, app developers must find the right balance by designing tools that limit passive screen engagement and encourage active participation. Transparent communication with caregivers about the purpose of the app as a facilitator of healthier habits, rather than a replacement for physical activity, remains critical. By promoting trust and addressing parental concerns, the app can serve as a valuable resource to support the health and well-being of children, adolescents, and young adults with cancer.

Given the wide range of cancer entities, a small number of patients were unable to use the app due to medical reasons, such as vision impairments or cognitive deficits. Despite efforts to develop a tool tailored to the needs of children, adolescents, and young adults with cancer, addressing the diverse diagnoses and associated limitations remains challenging. Future adaptations, such as audio feedback or caregiver guidance, could enhance accessibility and inclusion for this subgroup of patients, ensuring broader usability and engagement. A considerable number of participants did not provide further information on their reasons for not using the app. Given that the FORTEe trial is a multicomponent intervention encompassing exercise sessions, fitness assessments, questionnaires, video training, and other digital tools, the app was offered as an optional component with voluntary participation. This may have led some participants to perceive the app as supplementary rather than essential, which could have contributed to its lower engagement and limited feedback from nonusers.

Although the number of users was relatively small, their feedback still provides valuable insights into the effectiveness and acceptance of the “FORTEe Get Strong” app among children, adolescents, and young adults with cancer. Designed specifically for this population, the app received overall positive feedback for its design, user-friendliness, and interactive features such as the avatar and quiz. These elements likely played an important role in making the app engaging and appealing to young patients—an essential factor when addressing the challenges of motivating children to participate in physical activity and engage in other health-related topics [16,18]. Suggestions for additional features, such as a chess game or a virtual fitness studio, further highlight the patients’ preferences for more interactivity and variety. These findings provide valuable guidance for future app development, ensuring that the app becomes a more comprehensive and dynamic tool to support children, adolescents, and young adults with cancer.

However, certain areas of the app, particularly the exercise content and informational features, also received some less favorable ratings. This can be explained by the feedback from young users, who expressed a preference for supervised face-to-face training and the opportunity for more interaction. For example, several users noted that the exercises were less individually tailored compared with those provided during supervised training sessions, underlying the importance of supervision and personalization. Future versions of the app could address this gap by including a wider range of exercises with more adjustable difficulty levels to meet the diverse needs of users. Furthermore, the qualitative feedback about the app’s suitability across different age groups indicated that the target age range was defined quite broadly, ranging from 4 to 21 years. While some patients appreciated the app, particularly during hospitalization, some adolescents and young adults found it less engaging and felt it was better suited for younger children. However, Kruskal-Wallis tests of the Likert-scale ratings of specific app features showed no significant differences across the 4 defined age groups, suggesting that age does not systematically affect app ratings in this sample. As a result, the development of future digital health technologies should be tailored to the different developmental stages of users to better address their individual needs. Furthermore, the involvement of users throughout the development process can further ensure that an app effectively addresses the interests and needs of its target audience. Therefore, patient representatives were consulted on as many aspects of the app as possible, including specific design aspects such as the main theme and avatar design to make the app appealing to the target group. However, even when user preferences are taken into account, not everyone’s taste can be catered for, which may explain the feedback on age appropriateness.

### Limitations

A notable limitation of this study is that the evaluation was based on a relatively small sample (n=53) using and providing feedback on the app at the time of the assessment. As this evaluation was embedded within the larger FORTEe trial, the subgroup of app users was smaller than the trial’s overall sample, and no dedicated power calculation was performed for the substudy. Consequently, the study may have limited power to detect small differences between groups. As previously discussed, the small sample size in this investigation may be partly due to the comprehensive nature of the FORTEe trial.

Previous studies have shown that children are less likely to engage with apps that have a strong educational component and tend to rate them lower than scientific experts [37], which could have resulted in limited app use. Additionally, due to data protection regulations, the app usage duration was not recorded, resulting in limited insights into the frequency and consistency of engagement, as well as the interpretation of usage patterns and the relationship between intensity of use and perceived effectiveness. Similarly, adherence to the prescribed use of the app could not be objectively monitored; however, this might be critical to the app’s potential effectiveness. Therefore, future studies should consider incorporating self-reported adherence measures to better understand engagement behavior while maintaining compliance with data protection requirements.

Furthermore, although the app was conceptually grounded in SCT and SDT, the evaluation did not include explicit measures of theory-driven constructs, such as self-efficacy, outcome expectations, autonomy, and competence support. Consequently, it is unclear to what extent these mechanisms were activated. Future research should incorporate targeted assessments to empirically evaluate these theoretical processes.

Additionally, despite being designed for a broad age range (4‐21 y), some adolescents and young adults found the child-friendly design less engaging. Future iterations may therefore benefit from offering more age-differentiated design options to better accommodate diverse preferences across developmental stages.

Although efforts were made to minimize potential social desirability bias by encouraging participants to complete the questionnaire independently and limiting parental or staff assistance to clarification only, some degree of influence, particularly among younger children, cannot be ruled out. In future studies, this bias could be reduced further by systematically recording the presence of parents during completion of the questionnaire (eg, through a binary question about parental presence) and by standardizing the setting in which responses are collected.

Furthermore, while conducting the interviews in the native language of both the exercise professional and the participants likely improved the clarity and depth, this subsequent translation into English for documentation may have introduced information loss or translation errors. Additionally, some questions may have been difficult for younger children to fully comprehend, even in their native language, leading to potential misinterpretations or incomplete answers. These factors could affect the reliability and accuracy of the collected data.

### Conclusion

This study demonstrates that the “FORTEe Get Strong” smartphone app can support children, adolescents, and young adults with cancer in maintaining physical activity and health education. This study provides novel insights into user experience of children, adolescents, and young adults with cancer with a gamified exercise and health education app during intensive treatment, a phase that has largely been underrepresented in pediatric oncology mHealth research. Unlike previous digital interventions that focused primarily on survivorship, this mixed methods evaluation provides a more comprehensive understanding of how children, adolescents, and young adults interact with digital tools during intensive treatment. The findings contribute to the field by identifying key facilitators and barriers to engagement, including exercise variety, age-appropriateness, and the need for privacy-compliant peer interaction, thereby informing the design of future pediatric oncology mHealth interventions. From a clinical perspective, digital tools can help bridge care gaps when exercise professionals are unavailable by providing low-threshold access to exercise and health education, when in-person support from exercise professionals is limited, for example, during inpatient stays or periods of treatment-related isolation. These findings indicate that gamified digital interventions can facilitate continuity of exercise engagement, complementing traditional care across different settings. Overall, digital tools like the “FORTEe Get Strong” app have the potential to become an integral part of the comprehensive, multidisciplinary approach by empowering young patients with cancer to stay active, engaged, and supported throughout their recovery and beyond. Moreover, the insights gained from this study may be applicable to other pediatric patient populations with chronic conditions, suggesting broader relevance for the development of gamified digital interventions. Future studies should evaluate adherence, behavioral outcomes, and the app’s effectiveness in improving physical activity in larger samples to obtain a broader perspective on the implementation and impact of the “FORTEe Get Strong” app in the clinical setting of children, adolescents, and young adults with cancer.

## Supplementary material

10.2196/75653 Multimedia Appendix 1 Questionnaire / interview guidance of the FORTEe trial (English version).

10.2196/75653 Checklist 1 Mixed Methods Reporting in Rehabilitation and Health Sciences checklist.

## Abbreviations

mHealth: mobile health
SCT: social cognitive theory
SDT: self-determination theory

## References

[1] (2023). ECIR - European Cancer Inequalities Registry: Childhood cancer. European Commission.

[2] Cochran ED, Jacobson JC, Nehrubabu M, Qiao J, McCreery S, Chung DH (2024). Social determinants of outcomes disparity among pediatric patients with solid tumor. J Am Coll Surg.

[3] Erdmann F, Frederiksen LE, Bonaventure A (2021). Childhood cancer: survival, treatment modalities, late effects and improvements over time. Cancer Epidemiol.

[4] Braam KI, van der Torre P, Takken T, Veening MA, van Dulmen-den Broeder E, Kaspers GJL (2016). Physical exercise training interventions for children and young adults during and after treatment for childhood cancer. Cochrane Database Syst Rev.

[5] Stössel S, Neu MA, Wingerter A (2020). Benefits of exercise training for children and adolescents undergoing cancer treatment: results from the randomized controlled MUCKI trial. Front Pediatr.

[6] Fiuza-Luces C, Padilla JR, Soares-Miranda L (2017). Exercise intervention in pediatric patients with solid tumors: the physical activity in pediatric cancer trial. Med Sci Sports Exerc.

[7] Saultier P, Vallet C, Sotteau F (2021). A randomized trial of physical activity in children and adolescents with cancer. Cancers (Basel).

[8] Coombs A, Schilperoort H, Sargent B (2020). The effect of exercise and motor interventions on physical activity and motor outcomes during and after medical intervention for children and adolescents with acute lymphoblastic leukemia: a systematic review. Crit Rev Oncol Hematol.

[9] Morales JS, Valenzuela PL, Velázquez-Díaz D (2021). Exercise and childhood cancer-a historical review. Cancers (Basel).

[10] Braam KI, van Dijk-Lokkart EM, van Dongen JM (2017). Cost-effectiveness of a combined physical exercise and psychosocial training intervention for children with cancer: results from the quality of life in motion study. Eur J Cancer Care.

[11] Kauhanen L, Järvelä L, Lähteenmäki PM (2014). Active video games to promote physical activity in children with cancer: a randomized clinical trial with follow-up. BMC Pediatr.

[12] Mönninghoff A, Kramer JN, Hess AJ (2021). Long-term effectiveness of mHealth physical activity interventions: systematic review and meta-analysis of randomized controlled trials. J Med Internet Res.

[13] Boudreaux ED, Waring ME, Hayes RB, Sadasivam RS, Mullen S, Pagoto S (2014). Evaluating and selecting mobile health apps: strategies for healthcare providers and healthcare organizations. Transl Behav Med.

[14] Han M, Lee E (2018). Effectiveness of mobile health application use to improve health behavior changes: a systematic review of randomized controlled trials. Healthc Inform Res.

[15] Gu Y, Guan Y, Meng Z (2023). Health providers’ perceptions and experiences of using mHealth for chronic noncommunicable diseases: qualitative systematic review and meta-synthesis. J Med Internet Res.

[16] Baumann H, Fiedler J, Wunsch K, Woll A, Wollesen B (2022). mHealth interventions to reduce physical inactivity and sedentary behavior in children and adolescents: systematic review and meta-analysis of randomized controlled trials. JMIR Mhealth Uhealth.

[17] Schoeppe S, Alley S, Rebar AL (2017). Apps to improve diet, physical activity and sedentary behaviour in children and adolescents: a review of quality, features and behaviour change techniques. Int J Behav Nutr Phys Act.

[18] Ghosh P, Proffitt R, Bosworth KT (2024). mHealth app features that facilitate adolescent use for lifestyle management, and are endorsed by caregivers and health care providers. Mhealth.

[19] Neu MA, Dreismickenbecker E, Lanfranconi F (2025). Get strong to fight childhood cancer - an exercise intervention for children and adolescents undergoing anti-cancer treatment (FORTEe): rationale and design of a randomized controlled exercise trial. BMC Cancer.

[20] Bandura A (1986). Social Foundations of Thought and Action: A Social Cognitive Theory.

[21] Ryan RM, Deci EL (2000). Self-determination theory and the facilitation of intrinsic motivation, social development, and well-being. Am Psychol.

[22] Beauchamp MR, Crawford KL, Jackson B (2019). Social cognitive theory and physical activity: mechanisms of behavior change, critique, and legacy. Psychol Sport Exerc.

[23] Fuemmeler BF, Holzwarth E, Sheng Y (2020). Mila Blooms: a mobile phone application and behavioral intervention for promoting physical activity and a healthy diet among adolescent survivors of childhood cancer. Games Health J.

[24] Teixeira PJ, Carraça EV, Markland D, Silva MN, Ryan RM (2012). Exercise, physical activity, and self-determination theory: a systematic review. Int J Behav Nutr Phys Act.

[25] Edwards EA, Lumsden J, Rivas C (2016). Gamification for health promotion: systematic review of behaviour change techniques in smartphone apps. BMJ Open.

[26] Götte M, Gauß G, Dirksen U (2022). Multidisciplinary Network ActiveOncoKids guidelines for providing movement and exercise in pediatric oncology: consensus‐based recommendations. Pediatr Blood Cancer.

[27] Tovin MM, Wormley ME (2023). Systematic development of standards for mixed methods reporting in rehabilitation health sciences research. Phys Ther.

[28] Mellor D, McCabe M, Ricciardelli L, Ball K (2004). Body image importance and body dissatisfaction among Indigenous Australian adolescents. Body Image.

[29] Mellor D, Moore KA (2014). The use of Likert scales with children. J Pediatr Psychol.

[30] van Laerhoven H, van der Zaag‐Loonen H, Derkx B (2004). A comparison of Likert scale and visual analogue scales as response options in children’s questionnaires. Acta Paediatr.

[31] Wright KD, Asmundson GJG (2003). Health anxiety in children: development and psychometric properties of the Childhood Illness Attitude Scales. Cogn Behav Ther.

[32] Sawyer SM, Azzopardi PS, Wickremarathne D, Patton GC (2018). The age of adolescence. Lancet Child Adolesc Health.

[33] Mayring P (2014). Approaches to Qualitative Research in Mathematics Education.

[34] Gulec H, Smahel D (2022). Individual and parental factors of adolescents’ mHealth app use: nationally representative cross-sectional study. JMIR Mhealth Uhealth.

[35] Mateo-Orcajada A, Vaquero-Cristóbal R, Abenza-Cano L (2023). Gender and academic year as moderators of the efficacy of mobile app interventions to promote physical activity in adolescents: a randomized controlled trial. Humanit Soc Sci Commun.

[36] Reid Chassiakos YL, Radesky J, Christakis D, Moreno MA, Cross C, COUNCIL ON COMMUNICATIONS AND MEDIA (2016). Children and adolescents and digital media. Pediatrics.

[37] Wirth A, Mues A, Birtwistle E, Niklas F (2024). Evaluating educational apps for preschoolers: differences and agreements between the assessments of experts, parents, and their children. Comput Human Behav.

