# Supplementary material for: Development and Usability of the “FORTEe Get Strong” App to Promote Physical Activity and Health Awareness in Children and Adolescents With Cancer During Intensive Treatment Using an App-Based Approach: Mixed Methods Study
Source: JMIR Serious Games. 2026 Apr 1;14:e75653. doi: 10.2196/75653 (PMC13043004; doi:10.2196/75653)
Supplement: Multimedia Appendix 1 [file games-v14-e75653-s001.pdf]

Time of measurement (Please tick):	
☐ T0 ☒ T1 ☐ T2 ☐ T3 ☐ T4	
FORTEe ID:	Date:

Half-Structured Interview Physical Activity

Version for the Exercise Group

Version for partners not using Pixformance or Augmented Reality

T1

This project received funding from the European Union Research and Innovation Programme Horizon 2020 under Grant Agreement No. 945153.

**FORTEe ID:**	**Date:**

Instruction for the interviewer:

The main objective of the following interview is to obtain the perspective of the study participant concerning physical activity behavior and the FORTEe exercise intervention.

The following questions will guide the interviewer through the interview and **do not need to follow the exact same phrasing**. Thus, the interviewer should **adapt the formulation** of the questions to the language of the interviewee such as for a child, adolescent or adult.

The interviewer should also probe in order to dig deeper into the interviewees' responses through follow-up questions. The interviewee should be given the full attention to listen for their statements of facts, but also for their attitudes and beliefs.

The questions of the semi-structured interview at **T1 for the exercise group** relate to the patient's **physical activity behavior**, the **evaluation of the exercise intervention**, the **technology** used in the FORTEe project as well as the patients **personal experience in being physically active**.

Some of the questions need to be answered using the following **5-point scale** or **3-point-scale**. The **3-point-scale** should be used **for patients under the age of 8.**

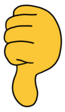	1 = Not at all	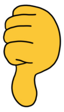	1 = Not at all
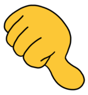	2 = Hardly		
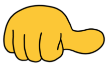	3 = Somewhat	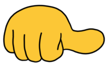	3 = Somewhat
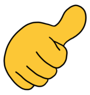	4 = Quite a lot		
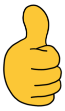	5 = Very much	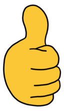	5 = Very much

FORTEe ID:	Date:

Physical Activity

	Not at all	Hardly	Somewhat	Quite a lot	Very much
How much do you enjoy being physically active/doing sports?	☐ 1	☐ 2	☐ 3	☐ 4	☐ 5
How physically active have you been recently (during the last week)?	☐ 1	☐ 2	☐ 3	☐ 4	☐ 5

What kind of physical activity/exercise have you been doing recently (during the last week)? *(please write down the name of the physical activities/exercises in the fields below)*	Please indicate on how many days during the past week you have been physically active/exercising and for how long.	
Physical activity/exercise No. 1:	______ days per week	______ min per day
Physical activity/exercise No. 2:	______ days per week	______ min per day
Physical activity/exercise No. 3:	______ days per week	______ min per day

**FORTEe ID:**	**Date:**

**What motivates you to be physically active/do sports?**<br>*(e.g. Why are you physically active? What do you like about it?)*

**Is there something that prevents you from being physically active/doing sports?**<br>*(e.g. Is there something you do not like about being physically active/doing sports?)*	☐ **Yes** ☐ **No**
If yes, please describe.	

FORTEe ID:	Date:

FORTEe Exercise Program

	Not at all	Hardly	Somewhat	Quite a lot	Very much
How much did you like the FORTEe exercise program?	☐ 1	☐ 2	☐ 3	☐ 4	☐ 5

What did you like most about the FORTEe exercise program?

Which training content/exercises did you enjoy the most in the FORTEe exercise program?

**FORTEe ID:**	**Date:**

**Was there something that you did not like about the FORTEe exercise program?**	☐ Yes	☐ No
If yes, please describe what you did not like and why.		

**Would you recommend the FORTEe exercise program to other patients?**	☐ **Yes**	☐ **No**
Why? Please describe.		

FORTEe ID:	Date:

Technology

Did you use the FORTEe webapp? ☐ Yes ☐ No
If no, why not? Please describe (e.g. not offered, no appropriate device, …)

If you used the FORTEe webapp, please rate how much you liked it.	Not at all	Hardly	Somewhat	Quite a lot	Very much
	☐ 1	☐ 2	☐ 3	☐ 4	☐ 5

FORTEe ID:	Date:

What did you like about the FORTEe webapp?

What did you not like about the FORTEe webapp?

FORTEe ID:	Date:

Did you use the FORTEe mobile app?	☐ Yes ☐ No
If no, why not? Please describe (e.g. not offered, no smartphone, not enjoyed…)	

If you used the FORTEe mobile app, please rate how much you liked…	Not at all	Hardly	Somewhat	Quite a lot	Very much
…the app.	☐ 1	☐ 2	☐ 3	☐ 4	☐ 5
…the design.	☐ 1	☐ 2	☐ 3	☐ 4	☐ 5
…the avatar.	☐ 1	☐ 2	☐ 3	☐ 4	☐ 5

FORTEe ID:	Date:

If you used the FORTEe mobile app, please rate how much you liked…	Not at all	Hardly	Somewhat	Quite a lot	Very much
…the exercises.	☐ 1	☐ 2	☐ 3	☐ 4	☐ 5
…the FORTEe quiz.	☐ 1	☐ 2	☐ 3	☐ 4	☐ 5
…the informational content.	☐ 1	☐ 2	☐ 3	☐ 4	☐ 5
…the user-friendliness.	☐ 1	☐ 2	☐ 3	☐ 4	☐ 5

Is there any comment that you would like to add about the FORTEe mobile app?

FORTEe ID:	Date:

Personal experience in being physically active

	Not at all	Hardly	Somewhat	Quite a lot	Very much
**Do you think being physically active/doing sports is important for your health and well-being?**	☐ 1	☐ 2	☐ 3	☐ 4	☐ 5
**Do you think being physically active/doing sports makes you happier?**	☐ 1	☐ 2	☐ 3	☐ 4	☐ 5
**Do you feel confident when being physically active/doing sports?**	☐ 1	☐ 2	☐ 3	☐ 4	☐ 5

FORTEe ID:	Date:

Further Questions for patients 12 years or older

	Not at all	Hardly	Somewhat	Quite a lot	Very much
Did the FORTEe exercise program help you to do something for your health on your own initiative?	☐ 1	☐ 2	☐ 3	☐ 4	☐ 5
Did the FORTEe exercise program help you to better cope with your disease?	☐ 1	☐ 2	☐ 3	☐ 4	☐ 5
Did the FORTEe exercise program make you more aware of your health problems?	☐ 1	☐ 2	☐ 3	☐ 4	☐ 5
Did the FORTEe exercise program help you to better cope with difficult situations?	☐ 1	☐ 2	☐ 3	☐ 4	☐ 5
Did the FORTEe exercise program help you to gain more confidence in your abilities?	☐ 1	☐ 2	☐ 3	☐ 4	☐ 5
Did the participation in the FORTEe exercise program make you feel more stressed?	☐ 1	☐ 2	☐ 3	☐ 4	☐ 5

FORTEe ID:	Date:

Further information and comments

If you would like to motivate another patient to join the exercise program during his/her anti-cancer treatment, what would you tell him/her?

Is there anything more you would like to add?
