# Supplementary material for: Development and Usability of the “FORTEe Get Strong” App to Promote Physical Activity and Health Awareness in Children and Adolescents With Cancer During Intensive Treatment Using an App-Based Approach: Mixed Methods Study
Source: JMIR Serious Games. 2026 Apr 1;14:e75653. doi: 10.2196/75653 (PMC13043004; doi:10.2196/75653)
Supplement: Checklist 1 [file games-v14-e75653-s002.pdf]

Mixed Methods Reporting in Rehabilitation & Health Sciences (MMR-RHS)

<u>Instructions:</u> The following checklist outlines essential information for mixed methods reporting. **1) Indicate "Y" if the standard is *fully met* or "N" and additional comments if lacking**. **2) Document page number where element is located**.

For more specific guidance, refer to the following linked checklists and individual journal requirements: Clinical Trials (CONSORT), Diagnostic Studies (STARD), Measurement Evaluation (COSMIN); Observational studies (STROBE); Intervention (TIDieR); Quality Improvement (SQUIRE); Qualitative (SRQR).

**Title**	**Y/N; Comments**
Concisely describes the topic of the study identifying the study as mixed methods	Y, p.1
**Abstract**	**Y/N; Comments**
Summarizes key elements using *journal specific* abstract format; For example: Introduction, Methods, Results, Discussion, and Significance/potential impact to rehabilitation and/or societal health	Y, p.1
	**Y/N; Page # Comments**
**Introduction**	
Includes literature review on the topic of interest (quantitative, qualitative, and mixed)	Y, p.2-3
Identifies gap that justifies the need for mixed methods approach	N
Clearly states overarching goal of the study that supports a mixed methods approach	Y, p.3
States the rationale for using mixed methods research	N
Clearly identifies discrete aim(s) for qualitative and quantitative components Aims align with corresponding component methods	Y, p.3
Provides statement of significance and potential impact	Y, p.2-3
**Methods**	
**Design –** *Clearly describes the mixed methods design* (exploratory sequential, explanatory sequential, concurrent, etc.) used to accomplish the overarching goal of the project:<br>• *Emphasis noted* (i.e., Sequential QUAL--> quan or QUAN--> qual; Concurrent QUAL + QUAN)<br>• *Visual display of overall design highlighting integration* (e.g., model, flow chart, figure)	Y, p.4-5 (Evaluation of the app)

© 2023 Tovin M. & Wormley M.

Mixed Methods Reporting in Rehabilitation & Health Sciences (MMR-RHS)

Methods (continued…)	Y/N; Page # Comments
Describes and supports the qualitative and quantitative methodologies (phenomenology, randomized control trial) used to accomplish the discrete aim(s) of the project	Y, p.6-7 (Data analysis)
States researcher(s) background and contributions to project (e.g. content or methods expertise, relationships to participants)	Y, p.5-6 (Method of assessment)
Identifies setting (e.g. hospital system, geographical location)	Y, p.4-5 (Study design; Participants)
**Subjects/Participants** - Clearly describes and supports the following:<br>• Sampling and recruitment<br>• Inclusion/Exclusion criteria<br>• Ethical considerations (consent process, researcher relationship with participants)	Y, p.5 (Participants; Ethical considerations)
**Data collection** - Clearly describes and supports the following:<br>• Pilot study (if applicable)<br>• Instrumentation (validity, reliability)<br>• Implementation matrix (e.g. data source, timeline, type, anticipated outcomes)	Y, p.4-6 (Study design, Method of assessment)
**Data analysis** - Clearly states and describes analysis procedures for:<br>• Qualitative<br>• Quantitative<br>• *Mixed Methods (integration)*	Y, p.6-7 (Data analysis)
**Methodological Rigor** – Clearly describes steps taken to establish rigor:<br>• Qualitative (e.g. credibility, dependability, confirmability, transferability)<br>• Quantitative (e.g. validity, reliability, generalizability)<br>• *Mixed Methods* (validity or legitimacy)	Y, p.6-7 (Data analysis)
**Results/Findings**	
Clearly presents findings for study components:<br>• Qualitative (includes data exemplars)<br>• Quantitative<br>• *Mixed Methods-Provides integrated findings/overall study results* (e.g., joint display)	Y, p.10-14 (Evaluation of the app)
**Discussion**	
• *Incorporates discussion on implications of integrated findings*	Y, p.15-19
• Provides synthesis and interpretation of findings in the context of existing literature and theoretical/conceptual framework	Y, p.14-19
• Includes subsection of limitations	Y, p.18-19 (Limitations)

© 2023 Tovin M. & Wormley M.
